# Exploring the Outcomes That Matter Most to Young People Treated for Chronic Pain: A Qualitative Study

**DOI:** 10.3390/children8121170

**Published:** 2021-12-10

**Authors:** Rhiannon Joslin, Maggie Donovan-Hall, Lisa Roberts

**Affiliations:** 1 School of Health Sciences, University of Southampton, Southampton SO17 1BJ, UK; mh699@soton.ac.uk (M.D.-H.); L.C.Roberts@soton.ac.uk (L.R.); 2 Women’s and Children’s Department, University Hospitals Sussex, St. Richards Hospital, Chichester PO19 6SE, UK; 3 Therapy Services Department, University Hospital Southampton NHS Foundation Trust, Southampton SO16 6YD, UK

**Keywords:** chronic pain, persistent pain, child, adolescent, outcome, qualitative

## Abstract

Global and national policies state that all children and young people should be part of decision making and that outcomes that matter to them should take priority, yet patient-centred outcomes have been identified as a gap in the paediatric chronic pain literature. This study gave youths experiencing chronic pain a platform to have their views heard. Using novel methods, twenty-one young people, aged 11 to 18 years old, completed a semi-structured interview in which they constructed a timeline drawing to symbolise their treatment. They identified when aspects of their life changed (outcomes) and described the importance of these changes. Thematic analysis identified four themes that emerged at different stages of the treatment: “perfect storm”; “turning points”; “disconnect”; and; “free”. “Turning points” were points in time when the narrative of the young person took a turn in a different direction. At these points, the outcomes important to them also changed. Youths initially prioritised outcomes related to pain, then during treatment the focus became their emotional functioning, with role functioning and “going out” becoming the focus at the end. The stage of treatment as perceived by the young person impacted which outcomes mattered most.

## 1. Introduction

Chronic pain affecting young people has been highlighted as a major health concern, with prevalence rates ranging between 11–38% [1]. In comparison to their peers, youths experiencing chronic pain have reduced function [2,3], poor sleep [4], reduced school attendance [5,6], and use more health services [7]. A third of young people seeking hospital treatment for chronic pain experience comorbid psychiatric and psychological conditions [8]. A multidisciplinary approach is consistently advocated as treatment. This focuses on functional recovery and includes common features of education, symptom control, psychological support, and physical therapy. However, despite intervention, chronic pain can persist into and throughout adulthood [3,8,9,10,11].

Outcomes from treatment can be both positive and negative. Guidelines exist that state which outcome domains should be measured in chronic pain research trials [12], but there is a gap in the literature when establishing and measuring patient-centred outcomes in clinical practice [13]. Moreover, existing studies that explored outcome preferences qualitatively found that adults with rheumatoid arthritis [14] and youths with neuro disability [15] identified outcomes that did not map onto an existing core outcome set or framework. Studies that have explored an outcome measure [16] or outcome preference [17] with youths experiencing chronic pain have aimed to gain consensus of opinion across patients, parents, healthcare professionals, and researchers. In doing so, outcomes identified as being important by youths were not always included in the final outcome measure or domain choices. One overlooked outcome appears to be health professionals’ understanding, a theme consistently highlighted in qualitative studies [18,19,20,21,22,23].

While there is evidence that physical functioning of youths improves over the course of multidisciplinary treatment, whereas pain intensity does not [24], it is unknown whether this physical change is important to young people or whether other outcome domains would best indicate a treatment is effective. Research trials prioritise measuring the four domains of: (1) pain intensity; (2) physical functioning; (3) emotional functioning; and (4) role functioning [25,26]. Studies compare baseline measurements to measurements taken at the end of treatment. However, an appreciation of subtle changes during treatment allows clinical approaches to be tailored to the individual, influencing adherence. Studies rarely examine changes over the treatment course, but some young people appear to make an independent decision to make positive change during treatment [23]. This shift in focus may change youths’ opinion regarding which outcomes are important, but this is yet to be established.

This study sought to gain the opinions of young people during their multidisciplinary treatment for chronic pain aiming to: (1) establish which outcomes young people identify as important and why; and (2) if their outcome preferences change during the course of treatment.

## 2. Methods

### 2.1. Study Design

A qualitative study using semi-structured interviews was conducted to investigate opinions regarding the changes (outcomes) youths may experience as a consequence of their chronic pain treatment. While all the interviews followed the same interview schedule (shown in Table A1), multiple media were offered (face-to-face at home or in hospital, telephone, online video, or online messenger), allowing those who felt unable to communicate verbally to take part. In addition, a participatory activity of drawing a timeline of their treatment was incorporated into the interview schedule. This allowed outcomes to be explored over time and gave the option to write and draw as a way of communication. Young people were purposefully sampled at different stages of their treatment and only completed one interview.

### 2.2. Participant Recruitment

Participants were recruited from two hospitals in England. Recruitment was carried out through local services (i.e., physiotherapy departments, rheumatology clinics and child psychology services) and tertiary multidisciplinary chronic pain services. This multifaceted approach aimed to elicit stories from youths who had not yet reached tertiary services, who potentially had made a full-recovery within a local service, as well as those who were more severely affected. Youths were eligible for the study if they were aged between 11 and 18 years old and were diagnosed with chronic musculoskeletal pain. It was defined as pain that was located in muscle, joints, or soft tissue; that had existed for more than three months; and where systemic disease (for example, juvenile idiopathic arthritis) that would otherwise account for the degree of pain had been excluded. Youths who had long-term comorbidities that had caused permanent disability and young people who were unable to express their views either verbally or in a written format in English were excluded.

Ethics approval was obtained from the National Health Service (NHS, Leeds, UK) Research Ethics Committee (Ref: 18/SC/0138). All eligible participants were initially invited by the clinical team by mail. Following this, where possible, participants were purposefully sampled based on age, gender, and treatment stage. Healthcare professionals approached young people who were at the beginning (within a month of initial assessment), during, or at the end of treatment (a month before or after the final appointment), until 6–8 youths were recruited within each stage (18–24 participants in total). This part of the recruitment was conducted face-to-face. On a monthly basis, the researcher visited the two clinical sites and updated clinical staff of the current gaps in recruitment. The researcher then attended clinics that were suggested by clinical staff that could potentially assist in recruiting a particular view-point. For example, a new patient clinic versus a follow-up clinic, or a clinic that had a larger number of older or younger youth. During this clinic, young people were given the opportunity to talk to the researcher about the study in a separate room. Ten youths who took part in the study had spoken to the researcher prior to their interview in these suggested clinics. Eleven youths had not met the researcher prior to their interview. They had received the study information by mail or face-to-face in a clinic where the researcher was not present. Forty-eight hours after receiving the study information, parents of youths who wished to take part contacted the researcher directly. Two parents reported their child did not want to participate. Young people provided their own informed, written consent. This was discussed with the Research Ethics Committee, which suggested Gillick competency could be applied. It felt that, for this study, it was important that parents were aware of their child’s involvement, but the youths felt empowered to give their own consent and agreed to be an active participant without coercion. Consent was revisited with youths prior to starting the interview.

### 2.3. Data Collection

Where possible, the young person was given the opportunity to talk without parental influence. It was established from patient involvement, at the design phase, that some youths felt unable to talk honestly in the presence of a parent as they were concerned that saying how they felt could increase their parents’ worry. During the interview, all youths were invited to complete a timeline drawing of their treatment from start to finish. For some youth, the timeline went into the future. Using the timeline, young people identified both positive and negative changes (outcomes) as a consequence of treatment and explained how these changes could indicate a treatment was effective or not. This drawing activity was designed and tested with patient involvement. Young people who chose to use the telephone or online methods of communication (video or online messenger) sent photos of their timeline at regular intervals via email or the messaging platform to allow the researcher to ask appropriate questions on the content.

The lead female author interviewed all participants and had extensive experience (over 15 years) working clinically as a physiotherapist with this group of young people at a different hospital site. The researcher was concurrently completing her PhD and was introduced to participants as a researcher. They were not involved in any of the participants’ clinical treatment. This was to ensure participation in the research study was separate to the young person’s treatment, and they could express their opinions freely without concern about it affecting their ongoing care. Immediately following the interview, the researcher documented field notes to capture non-verbal data, observations and reflections on the potential influence of the environment, presence of parents and/or impact of their involvement, and certain questions or probes. Interviews completed on a messenger service were automatically transcribed, whereas the audio from the other interview media were recorded and transcribed verbatim. The transcripts were not returned to participants, but all participants kept their timeline drawing outlining what they had discussed. A photograph copy was provided to the researcher for analysis. Any identifying information and/or unique traits that could lead to deductive disclosure were removed; this included removing age alongside the pseudonym on example quotes. Pseudonyms were chosen by the participants.

### 2.4. Data Analysis

The data were analysed and reported using the consolidated criteria for reporting qualitative research (COREQ) [27]. Although the timeline drawing was a participatory activity and, therefore, does not have a specific analysis [28], combining the interview and timeline data allowed interpretation over time. To achieve the study aims, thematic analysis, as outlined by Braun and Clarke [29], was used to allow the full narratives to be analysed, identifying similarities and differences between participants. Thematic analysis allowed the description of themes using the words of young people; therefore, the information could be accessible to a range of audiences, including the youths themselves. To establish whether opinions changed over the course of the treatment journey, the timelines were analysed first, which allowed initial codes to be linked to specific points along the treatment course. During the analysis of the final three interviews, no new initial codes were being identified, and the three authors felt the data had reached a point where further interviews would not add to the overall story.

Initial codes represented important data surrounding the topic of outcomes, such as “pain”, “sleep”, “getting a diagnosis”, and “mood”. The interview transcripts were then analysed and any new initial codes were added. This initial coding was applied to the first timeline and transcript by R.J. and L.R., together, to ensure the data could be captured. Following this, R.J. coded the timelines and transcripts continuing the same method. New initial codes were discussed with M.D.-H. and L.R. The initial codes were then collected onto a single timeline to enable the researchers to identify which initial codes held relevance through the treatment journey and which only held relevance at specific points. Separate drawings were then made for different initial codes, and the researchers explored how the initial codes and the language used to describe them changed over the treatment journey. For example, for the initial code, ‘pain’ language changed from “*uncontrollable pain*” at the start, to pain “*spread*” during treatment and “*pain free*” at the end of treatment.

The following stage involved examining all initial codes individually and creating an initial mind-map. This enabled the initial codes to be placed on a location within the timeline but also connections could be drawn between the codes through timeframes. On completion of the mind-map, the information was grouped together through discussions with the research team. The groups then developed into themes. The themes were discussed and re-evaluated until each theme was fully described. The themes and findings were shared with participants.

## 3. Results

Twenty-one youths volunteered and took part in the study between May 2018 and April 2019. Table 1 shows participant demographic information. Twelve participants (57%) were recruited from a tertiary service, and nine participants (43%) were recruited from either a physiotherapy service (n = 8) or psychology service (n = 1). Participants discussed receiving treatment in twelve different hospitals supported by five different tertiary services. Four participants chose to have a parent present during their interview, and one parent was intermittently present during the interview in the family home. Four of these five participants represented the youngest recruited (age 11–12 years old). The average length of interview was 54 min (range 35–98 min). According to the judgement of healthcare professionals, seven participants were considered as being recruited at the ‘start’ of treatment, nine ‘during’ treatment, and five at the ‘end’ of their treatment. However, from the viewpoint of participants, only two were at the start of treatment, and the majority placed their current stage as ‘during’ treatment. This discrepancy existed because participants linked previous pain experiences to their current situation, whereas healthcare professionals had not made these links.

### 3.1. Trajectories of Pain and Symptoms along the Timeline

Pain was most often described as “*hurt*”. During treatment, pain “*spread*”, and this was described in different ways: (1) as an extension of the existing pain; (2) additional joint pain as a consequence of using walking aids; and (3) as pain that moves round the body with or without resolution of one pain before another. None of the participants described fewer painful locations during treatment. The frequency of reported symptoms also increased during treatment and included fatigue/tiredness, dizziness, headaches, abdominal pain, loss of concentration, and “*episodes*” that included blackouts, loss of sight, and loss of sensation, without medical explanation. The only symptom that appeared to resolve during treatment for two participants was disturbed sleep.

At the end of treatment, pain and symptoms resolved but did so quietly, with participants making statements, such as “*it must of got better because I’m doing more*”. When asked specifically whether “*if we spoke at a different point of treatment*, *e.g., 6-months ago, would you have said anything different?*”*,* thirteen participants (62%) said they would have highlighted different outcomes as being most important at the beginning. For example, some “*wouldn’t have mentioned the mood*” or “*impact it has had on my family*” as they either had not realised the impact it had on their emotions or had chosen to keep these feelings to themselves. Instead, they would have focused on the pain and their physical ability.

### 3.2. Themes

**Figure 1:**
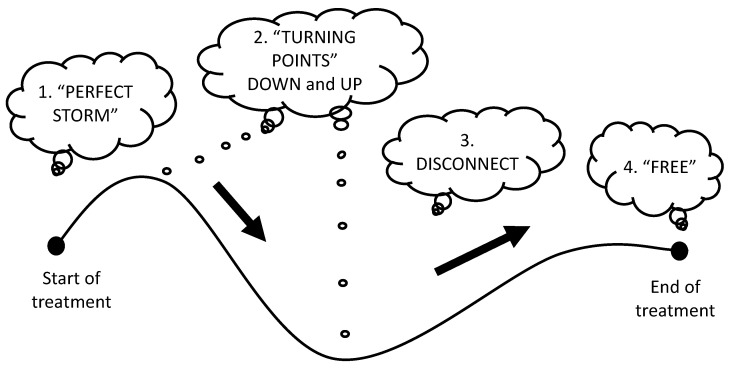
Four themes across the treatment journey.

Four themes emerged at different points of the young person’s treatment journey, as shown in Figure 1. These are outlined individually. In addition, a rollercoaster metaphor explains the underpinning storyline that forms the basis of these complex and personal stories about the treatment journey (Appendix B).

#### 3.2.1. Theme 1: The “Perfect Storm”

At the start of treatment, narratives focused on the outcome domain of pain. The theme was named from Harper’s transcript: “*I had like a perfect storm which caused the pain*”. Similar to a storm, pain arrived as a shock, it was an uncontrollable force, with uncertain consequences. Looking back, participants could see multifactorial reasons why the pain had started, akin to a storm building power. Recent bodily changes were described by the majority of participants and included: (1) changes to their thoughts and mood; (2) pubertal changes and growth; and (3) a traumatic event or bodily insult. Five participants discussed social changes including moving school, bullying, or more subtle friendship changes. Only one participant reported not having this build-up and felt the pain came “*out of the blue*”.

When the pain started, the language of participants implied it had an external power and had the ability to “*stop*” or “*restrict*” them from doing the things they wanted. They used words that suggested loss of choice—“*couldn’t*” or “*had to*” or “*unable*”. Only two participants suggested they chose how to respond to their symptoms using language, such as “*I don’t want to eat*” or “*I was quite preventative [sic]*”. The common narrative at this stage was uncertainty, as they felt healthcare professionals were confused by their symptoms, and repeated medical investigations did not explain the pain.

The loss that occurred from the pain was described as that of damage from a storm. Being isolated and lonely was most prominent in these narratives (n = 20). Mia wrote on her timeline at the start: “*no friends*” and “*alone*”. Most participants described losing friends or withdrawing themselves from friendships. In addition, physical loss, such as losing the ability to walk or move their body was described, as well as the loss of normal bodily functioning, with participants describing themselves as “*unwell*” or *“ill”*. Over half the participants described a wider influence on their family, most notably increased worry by their mothers.

*Riley: “I think my mum was quite worried. She thought, she told me, she thought that I, it might be like forever and I’d never be able to walk again”.*

#### 3.2.2. Theme 2:“Turning Points”

**Figure 2:**
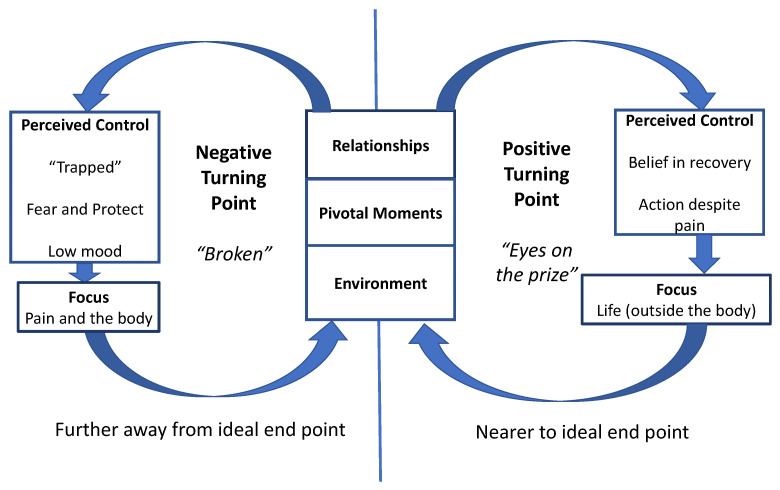
Summary of theme “turning points”.

“Turning points” were specific points in time when the narrative of the participants changed and took a different direction, either positive or negative. The theme was named from Abigail’s transcript and is summarised in Figure 2.

*Abigail: “I mean I was a bit more motivated to get better cos I didn’t know if I could get better. So, I suppose I was like, think it was a turning point, and I was like ‘I can do this’”.*

A positive “turning point” took participants closer to recovery, as described in the final theme “free”, whereas a negative turning point took them back to the “perfect storm”. It was during treatment when the outcome domain of emotional functioning became important. Belief in their own recovery was key, yet participants only believed they could recover when they perceived they were in control of their situation and made the required behavioural changes that focused on life (positive turning point). Conversely, when participants described a negative turning point, their perceived control was lost, they lost belief in recovery and protected themselves: their pain and their body became the focus. The change in perceived control was reflected in the language used by participants, changing between “*pain stopped me*” and “*I couldn’t*” versus “*I want to*”, “*I can*”, and “*I’m not going to let it stop me*”.

Worsening mental health was described by the majority of participants with words, including “*sad*”, “*depressed*”, and “*low*” written at the bottom of a negative turning point. One young person reported a previous suicide attempt, two reported suicidal ideation, one reported self-harm, and five reported they had been given a clinical diagnosis of depression during treatment. Quotes from participants at this point portrayed hopelessness about their situation and included: “*I had enough of everything*”, “*I’m basically a walking corpse*”, and “*I didn’t want to be alive*”. A small group also discussed worsening anxiety at this point, which included having panic attacks before appointments and “*worry about everything*”.

From the collective narratives, three factors facilitated a positive or negative turning point: (1) relationships; (2) the environment; and (3) pivotal moments. These sub-themes are discussed separately.

##### Sub-Theme: Relationships

Key qualities of relationships (with family, friends, school teachers, sports coaches, and healthcare professionals) that could facilitate a turning point included:

- Being “*connected*”—a positive relationship. When the relationship focused on them as a person *(*“*me*”*),* what they enjoyed, and had achieved (the “*positives*”). The other person understood their point of view and wanted to help. The young person felt included and accepted.
- An “*outsider*”—a negative relationship. The relationship focused on how they were different, their pain, the body part that hurt, or the equipment they used. The young person’s point of view was dismissed or over-powered by the other person. The young person felt alone and isolated.

When developing a relationship with healthcare professionals, participants valued trust, time to develop the relationship, and familiarity with chronic pain. Peer relationships were the only relationship that enabled the participant to feel included, accepted, and liked, so they no longer felt “*alone*”.

##### Sub-Theme: The Environment

Positive turning points were associated with participants interacting with a range of places, people, and objects outside the home. They had their own independence, made their own way to school and were “*going out*” with friends. When they spoke of their hospital treatment, they highlighted when physiotherapists or occupational therapists had taken them to the hydrotherapy pool, the gym, for a walk in the grounds, or to a local shopping centre. They described these sessions as “*fun*” and “*relaxed*” contrary to the 1:1 hospital appointments that they described as “*annoying*” and “*a hassle*”.

*Evelyn: “They said we could like do Pilates and go in the pool…not just doing stuff at home. Making it fun”.*

Conversely negative turning points were described by participants as “*at home all the time*”. Walking aids and wheelchair provision were given to increase access; however, the opposite effect was described. This included not being able to access their bedroom, being fearful of leaving the house or busy places, feeling threatened at school due to being called “*cripple*” and “*faker*”, and having more time off school. Within school, these participants spent the day in a library or inclusion centre to avoid crowds and stairs, they spent break or lunchtimes indoors, frequently attended the medical room, and/or did not attend their usual classes. Even transport to and from school became reliant on parents reducing the young person’s independence.

*Hannah: “…cos a lot of my classes then were upstairs and the school wouldn’t let me upstairs on crutches, so I spent a lot of my time sat in the library”.*

##### Sub-Theme: Pivotal Moments

Pivotal moments were events in a participant’s life that acted as a catalyst for change. These included an interaction with a healthcare professional, being admitted and discharged from hospital, making a disclosure, school transition points, holidays, and events outside the hospital, such as gaining a sports award (positive) or a family bereavement (negative). The most frequently reported pivotal moment was having their pain explained (diagnosis) by a healthcare professional. This could have both a positive and negative effect as outlined.

- Positive explanation of the pain. Their pain was taken “*seriously*”. The explanation “*made sense*” was relevant to their situation and existing beliefs. It was delivered with certainty and consistency by a trusted healthcare professional who had treated people such as them before. They felt justified there was “*something wrong*” that was “*reversible*”.

*Isabella: “I was really happy that day because I finally figured “I wasn’t making it up” and there was a reason for me getting upset”.*

- Negative explanation of the pain. Their pain was dismissed as “*normal*” or blamed on other factors, such as being “*overweight*” or “*anxious*”. The explanation made no sense and did not fit with their existing beliefs. It was delivered by a healthcare professional unfamiliar with their condition and/or was inconsistent between healthcare professionals. Discussion around psychology was interpreted as the pain was not real. They feared serious illness, sought further medical reassurance, and felt like pain was inevitable.

*Lily: “It’s just like this thought in your mind that you won’t get over it.”*

Hospital admissions that were described as negative pivotal moments included those that made symptoms worse, where they felt “*pushed*” or lost trust in their medical team. Conversely, when a young person felt safe in hospital or when the rehabilitation was tailored to their needs, participants described their admission positively. On discharge home, improvements were difficult to sustain and young people described feeling abandoned by healthcare professionals.

*Chloe: “I really did just want [the consultant] to see us…but there was nothing, there was nothing at all”.*

During or immediately after a hospital admission, three participants reported they made a disclosure. Two participants disclosed feelings of low mood and suicidal ideation, and one participant disclosed a recent traumatic event. These three young people describe this initial disclosure as making them lower in mood, “*fragile*”, and “*downest I’ve been so far*”. However, after speaking to a psychologist or representative of the mental health service, their parents and other agencies, making a disclosure appeared to be liberating and participants described feeling as if they could put their energy into doing things they wanted.

Holidays and school transition points (change of school or school year) offered participants an escape. Prior to going on holiday, Aria was unable to weight bear, Harper had not left the house, and Sophie was struggling to walk due to dislocations. Their holidays had transformative effects on their life: they managed physical challenges, such as climbing, going on waterslides, and jumping off a waterfall. While school transition points were largely positive, and participants described finally feeling as if they *“fit in*”, Alexander and Emma described the opposite effect when they moved from a school where they felt safe to one which they felt vulnerable.

### 3.3. Theme 3: Disconnect

All the participants could describe an ideal end-point of treatment when they would no longer require hospital appointments; however, a small group of participants could not visualise themselves “*getting better*”. Two of these youths left a gap on their drawings between where they were and their ideal end-point and this ‘disconnect’ in the line, was how the theme was named. Another two participants labelled the end-point as “*their whole life*” or described how recovery was “*never going to be possible*”. As well as a disconnect with recovery, some of these participants discussed a disconnect with their own body. They felt their body was irreversibly “*broken*”; it no longer felt as if it were their own, they were “*angry with it*”*,* and wanted an amputation or were “*blocking*” the body part. Two participants described this period of disconnect in their past. Psychology sessions had given them the optimism and belief that they could “*get better*” and highlighted this disconnect was subject to change.

### 3.4. Theme 4: “Free”

At the end of treatment, participants wanted to feel “*free*”. Here, the outcome focus became social functioning, primarily “*going out*” with friends. They wanted that feeling of escape, the ability to live their life with child-like spontaneity, a “*normal*” existence, where pain no longer had an external hold. “*Going out*” required a reason to leave the house, someone to go with, and a place to go. Being “*free*” also involved being “*pain free*”, having “*physicality*” back, and being “*happy*” and “*well*”. These factors were not viewed in isolation, and participants explained how pain and their mood “*factor in together*”. Emotionally, as well as “*happy*”, participants wanted a sense of being “*relaxed*” and “*carefree*” where they had the “*get up and go*” to just “*do it*”. Physically they described being able to walk, run and move their body without dislocations or joints making “*clicking*” or “*grinding*” noises. Being “*well*” meant they wanted to be able to eat regular meals, sleep consistently at night, not need “*pills*”, and have energy to do things. Lastly, a group of participants wanted to “*free*” their family, for their family to be “*happy*”, and their parents to have “*peace of mind*”.

## 4. Discussion

At the beginning of treatment, pain was the focus. Pain had control and was perceived to be the cause of many losses in a young person’s life. During treatment, emotional functioning took priority, but, without this being addressed, youths felt trapped and disconnected from their own recovery. Yet, if a positive turning point could be facilitated, recovery was visualised, and youths felt empowered to change their behaviour. When able to make these changes, their social life and “*going out*” with friends was their focus, and ultimately led to their recovery. Pain was the last thing to resolve.

The current study demonstrated that outcome preferences changed during the course of treatment and were dependent on the young person’s perceived stage of journey. This is a new discovery not yet established in the paediatric pain literature. However, in other adolescent populations, this dynamic and fluctuating understanding of recovery had been identified and differed from adult literature [30].

“Turning points” during treatment appeared pivotal to a young person’s recovery, yet research trials [25,26] and clinical practice rarely measure outcomes at regular points during treatment and potentially miss these fluctuations, or capture and analyse them without context. The treatment experience appeared key to facilitating change, but it is an outcome domain that is rarely measured [12,31].

What is concerning is that, during treatment, young people described a drop in their mood, and, for some, this had serious consequences. When considering reasons for this change in mood, one explanation is the unmasking of emotional and psychological distress when physical functioning improves. Somatic symptoms in adolescents have been found to precede depression and predict severe adult mental health disorders [32]. Another consideration is that the isolation from friends at the start could be a factor, as peer loneliness can be a predictor of depressive symptoms in adolescents [33].

Supporting the “turning point” theme, a qualitative study found eight young people made a decision to recover within 6–12 months of the start of their multidisciplinary treatment [23]; however, factors that facilitated this change had not been alluded to. Perceived control and a belief in recovery appeared central to the “turning point” theme and link to self-efficacy [34]. In the context of this study, self-efficacy describes the confidence young people had in their own ability to successfully function, despite pain. Self-efficacy had been identified as an important outcome by paediatric pain service stakeholders [17], and a topical review suggested self-efficacy could be a resilience mechanism in chronic pain [35]. While limited to those with chronic headache, there is also evidence that there is a strong relationship between self-efficacy and functional disability [36].

**Figure 3:**
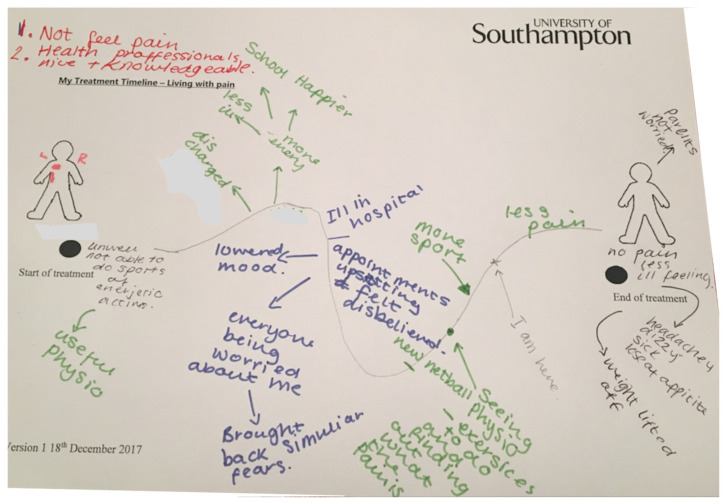
Norah’s timeline drawing to highlight similarities to the Kubler-Ross Change Curve [39].

Contrary to positive turning points, negative turning points are yet to be discussed in the literature. Only recently the unexplored detrimental effects of multidisciplinary treatment were established through a similar timeline methodology [37]. When exploring the literature more widely, the negative turning points, as drawn by youths and shown in Figure 3, resembled the Kubler-Ross Change Curve [38] that was expanded to describe the response to loss [39]. The names of the stages have changed over time; however, ‘depression’ occurring at the bottom of the curve after a gradual decline of morale and confidence reflected what had been written and drawn by the young people on their timelines. Interestingly, after the stage ‘depression’, the Kubler-Ross Change Curve [38,39] has a U-turn, and the following stages include ‘decision’ and ‘integration’, which have similarities to the descriptions of positive turning points and the theme “free”. This could suggest that low mood may be a stage that is experienced before recovery. Norah’s timeline (Figure 3) demonstrates this pictorially. The negative outcomes are written in blue on the downward curve and include being “*ill*”, “*fears*”, “*worried*”, “*disbelieved*”, “*upsetting*” and, “*lowered mood*”; then, positive outcomes are written in green on the upward curve, including “*finding out what the pain is*”, “*exercises to do*”, “*more sport*”, and “*less pain”*.

Other change theories using similar models have been used to describe the transition from child to adult [40]. This introduces the idea that youths are already experiencing emotional responses due to their stage of life with middle adolescence (age 15–17 years) being identified as a time of ‘storm and stress’ and turmoil in comparison to the restorative period of later adolescence [41]. This raises the question of how much does the transition into adulthood factor into the turning points and outcome priorities of youth?

Throughout these subthemes, the importance of feeling safe and a sense of belonging, shone through the narratives. Maslow’s Hierarchy of Needs theory of human motivation [42] would suggest that the lower order needs of safety, love, and belonging need to be met before addressing the higher order needs of acceptance, confidence, self-efficacy, and achievement (self-actualisation). This theory reminds us that if the lower order needs are not being met, this decision to change may feel unachievable for youth. The current study gave insight into what outcomes mattered to youths when having treatment and, in doing so, provided valuable information on factors that facilitated change. Having awareness of both outcomes that are important, but also why they are important in terms of change and recovery, can further improve clinical care for this vulnerable group of youths.

### 4.1. Limitations and Future Research

The opinions of youths represent experiences in health services in England and may not reflect the experiences of those using different hospital services or the experiences of youths in different countries. Future research could investigate the opinions of youths in countries where there are known differences in services and culture because the way a person interacts within their world may influence their outcome preference. The current study involved seeing youths in different settings and as highlighted in the reflections of the researcher, the interaction with the researcher and parents if present, may have influenced the responses. While the study sought to purposefully sample youths at different stages of their journey, the arbitrary time frames did not correspond with the stage of journey as perceived by the young person. In addition, it required participants to recall events in the past, increasing the risk of bias. In future research, outcomes of treatment could be aligned to timepoints poignant to the young person. Male youths were fewer in number and reflected the reduced incidence of young males experiencing persistent pain. This study did not aim to compare gender responses; however, this could be something to explore in future studies. Lastly, while every effort was made to include representatives of all youths seeking health service treatment for persistent pain, participants were voluntary responders, and one participant suggested they would have felt unable to participate at an earlier time-point due to low mood. The results of this study and other studies may, therefore, reflect the views of young people who have already sought help for depression/low mood or have yet to be affected from these difficulties. Research must, therefore, consider capturing the times that youths feel unable to participate in studies due to their low mood and consider how best to support them to express their opinion when they feel able to.

### 4.2. Clinical Implications

#### 4.2.1. The Context in Which the Treatment Was Delivered Mattered

Youths described nine different physiotherapy treatments, five psychological treatment approaches, eleven different medications (pharmacology), four complementary therapies, and one dietary treatment. However, not a single treatment emerged as a key component to a positive or negative turning point. Conversely, the way in which the treatment was delivered did matter.

#### 4.2.2. Listen to the Language and Expression of Young People and Tailor the Support

Language and expression within the young person’s narrative indicated where they were on their journey. Their words could suggest they had decided to take control of their situation but also when they had lost their perceived control. Healthcare professionals should reflect on their own influence and monitor outcomes of the treatment experience, as these become increasing important. Equally, a young person’s mental health should be carefully monitored, especially when they appear to be on the downward slope of a negative “turning point”.

#### 4.2.3. Giving a Diagnosis Is Not a Tick-Box Exercise

After a young person had been given an explanation for their pain that validated something was wrong and could recover, it facilitated youths to engage in a process of sense-making. The young person started to make their own mind-body connections and over time reflected on these with their healthcare team. Any change in symptom or new system had the potential to send the young person into the uncertainty of the “perfect storm”, yet, if the explanation of the pain remained consistent and was repeated with certainty, the process of sense making continued towards a belief in recovery.

## 5. Conclusions

The stage of treatment as perceived by the young person impacted upon which outcomes mattered most. Outcomes identified by youths were not seen in isolation; they were complex and interconnected. The current study highlighted the value of measuring outcomes during treatment. It has been demonstrated how youths prioritised outcomes relating to the treatment experience during the treatment process, and, if the world around them was “*right*”*,* they appeared to have the motivation required to make the decision to recover. However, these complex interactions between people, places, and objects are not easily measured. Youths expressed the importance of being happy, pain free, and “go out” (positive in focus). Multidisciplinary treatment outlines the importance of shifting the focus to wellness, yet the outcome measures currently used are contradictory focusing on pain, depression, and disability (negative in focus). Healthcare professionals and researchers need to consider how they tailor treatment to the individual, how they show young people a link from where they are now to where they want to be. Youths want to achieve the outcome ‘to believe I can get better’, and healthcare professionals can facilitate this belief, but also take it away. Parents and healthcare professionals need to remember the young person is the author of their own story, they hold the pen, and they chose how their story will end. Measuring outcomes at the start and end of treatment is akin to reading the beginning and end of a book; the meaning is lost, the twists and turns are missing, and the sparking events remain a mystery.

## Figures and Tables

**Table 1:** Demographic data of the participants (n = 21).

Characteristic	Results
Age in years (mean, range)	14.8 (11.11–18.0)
Gender n (%)	
Female	18 (86%)
Male	3 (14%)
Locations of Chronic Musculoskeletal Pain ^1^ n (%)	
Lower limb	12 (57%)
Multiple joints	6 (28%)
Neck, back and chest	2 (10%)
Upper limb	1 (5%)
Duration of treatment in months ^1^ (mean, range)	30 (2–78)
Duration of pain in months ^1^ (mean, range)	40 (5–108)
Treatment setting ^1^	
Only outpatient treatment	16 (76%)
Involved inpatient rehabilitation	5 (24%)
Precipitating events ^1^	
No event	11 (52%)
Injury	6 (29%)
Infection/illness	2 (10%)
Surgery	1 (5%)
Chronic disease	1 (5%)

^1^ Based on report from the young person.

## Data Availability

As this is qualitative research raw data will not be shared.

## Appendix A. Semi-Structured Interview Schedule

**Table A1:** Broad and probing questions.

Broad Question	Probing Follow-Up Questions
Looking at the empty timeline can you first go to the “START” point. 1. Can you tell me about yourself at the start of your treatment when you first came to hospital for pain treatment?	What was your pain like? Where did it affect? What were you able to do? How did you spend your time? What were you saying? How did you feel?
Now go to the “END’ point 2. At the END, describe yourself at the end of an IDEAL treatment when you no longer need to come to hospital. (This may be in the future.)	What would you be doing? Where would you be spending time? What would you be saying? How would you be feeling? What would your parents and family say?
Joining the dots 3. Now consider your journey from the start of treatment until the end and join the “Start” and “End” dots to show what your journey has looked like so far and what it might look like into the future. 4. Now mark the line with the point where you are now on this journey and write “I AM HERE” and an arrow.	Add any important events that have happened or are happening in your life.
Positive Signs 5. How would you know if a treatment is working? Write positive signs on your treatment timeline.	What are the early signs/ final signs a treatment is working? What changes make the biggest difference? Do you think health professionals/ parents notice these changes? Tell me more about x that you mentioned? Can you tell me more about why you chose these.
Negative signs 6. How would you know a treatment was not working? Draw negative signs on your treatment timeline.	What are the early/late signs that tell you treatment is not working? What changes cause the most impact and would stop you continuing the treatment? Do you think health professionals/ parents notice these changes? Tell me more about x that you mentioned? Can you tell me more about why you chose these.
Change over time 7. If we spoke at the start of treatment would you have said anything different?	

## Appendix B. The Final Metaphor-The Rollercoaster Journey—A Search for Light

At the start, you feel trapped by the rollercoaster, the environment is unfamiliar and the structure which is there to protect you is heavy and restraining. Although you are surrounded by other people you are segregated and while others are on this rollercoaster with you, they are not in your seat and have a different viewpoint and experience. You feel alone and scared on this journey. You may have expectations or hopes for what is to come, but ultimately you have no idea what is going to happen next and how it will make you feel. The knowledge that there is an end-point to this experience becomes critical. The vision of an end-point is like a light at the end of a dark tunnel, knowing you will be let free from this is like a driving force inside you.

Along the journey, there are twists and turns, progressive climbs and fast descents. Sometimes, there are such highs, where the light is so bright that the end-point is crystal-clear and you can look at your world from a whole new perspective. Equally the rollercoaster can come crashing down and take you to dark places where the vision of an end-point gets smaller, blurred and then invisible. You concentrate on protecting yourself but this decision traps you in an even smaller space. Feeling lost and in darkness you bravely reach to form connections with people who are near you. You are no longer alone on the journey. You look for light in a new corner of your environment, you take a chance, let go of your firm grip and reach your arms in the air, you scream in excitement, realising enjoyment is possible. With rollercoasters full of unexpected surprises, any moment your situation can change but you realise you have control over what happens next. You re-join the journey with a different attitude and somehow by focusing on those connections with people, those hidden corners of your environment and those events that shift your thinking, you feel strong and believe you can do this.

As you near the end, warmed by the light, you feel different. The ride becomes fun and the heaviness of the metal around you starts lifting without you realising, you are freed. At the end of the rollercoaster, you are lighter but unsteady, as you regain your energy and thoughts, you smile realising how far you have come and what an achievement it has been. It has been a tough journey but one which has made you appreciate the freedom which you now have. You can do what you want, when you want and have the childlike spontaneity back in your life. You look around and you walk off the rollercoaster with familiar smiling faces. They have been on the ride with you, they excitedly talk to you and you realise how important these people are. You talk back with ease and it feels like you belong.
